# Supplementary material for: Content-rich biological network constructed by mining PubMed abstracts
Source: BMC Bioinformatics. 2004 Oct 8;5:147. doi: 10.1186/1471-2105-5-147 (PMC528731; doi:10.1186/1471-2105-5-147)
Supplement: Additional File 2 — The original results of the above study (non-essential files are deleted to keep the file size under the limit set by BMC bioinformatics). [file 1471-2105-5-147-S2.bz2 › chilibotAdditionalFile2/dip20/424ID11470772E10354/html/CCL1_CCR8.html]

 


 **CCL1** and **CCR8** 
  
Found 39 abstracts in PubMed, retrieved 20.  
 

 What does Google say? 
 PDF only 
| .edu only 

---

**Interactive relationship** (e.g. stimulation, inhibition, etc)

**Neutral relationship**- Further analysis demonstrated that  **CCL1**  activates the MAPK pathway in  **CCR8**  transfected CHO cells.  Ref: 12645948 Eur J Immunol, 2003
- ... interactions of CCR4 with MDC CCL22 or TARC CCL17,  **CCR8**  with I 309  [ **CCL1** ]   **CCL1** , and CRTH2 with prostaglandin D 2 play a critical role in the allergen induced recruitment of Th2 cells in the target tissues of allergic inflammation.  Ref: 12009564 Mol Immunol, 2002
- Th2 cells preferentially express  **CCR8**  and migrate selectively to its ligand, CC chemokine ligand CCL 1  [ **CCL1** ] .  Ref: 12707363 J Immunol, 2003
- **CCR8**  is the sole receptor for the human CC chemokine I 309  [ **CCL1** ] , as well as for viral monocyte inflammatory protein I vMIP I, a human chemokine homologue induced in human cells by the Kaposi sarcoma related human herpesvirus 8.  Ref: 11133740 Blood, 2001
- The role of CCRS was supported by the observation that vMIP I, another  **CCR8**  ligand identified from the genome of a T cell transforming herpes virus, shared  **CCL1**  anti apoptotic activity.  Ref: 12645948 Eur J Immunol, 2003
- **CCR8**  is detected at later time points than CCR4 and exclusively on differentiated Th2 cells as revealed by analysis of mRNA expression and responsiveness to  **CCL1** .  Ref: 11981813 Eur J Immunol, 2002
- In vitro polarized human Th2 cells preferentially express the chemokine receptors CCR3, CCR4, and  **CCR8**  and migrate to their ligands eotaxin, monocyte derived chemokine MDC, thymus and activation regulated chemokine TARC, and I 309  [ **CCL1** ] .  Ref: 11390417 J Clin Invest, 2001
- Treg cells specifically express the chemokine receptors CCR4 and  **CCR8**  and represent a major subset of circulating CD4 T cells responding to the chemokines macrophage derived chemokine MDC CCL22, thymus and activation regulated chemokine TARC CCL17, I 309  [ **CCL1** ]   **CCL1** , and to the virokine vMIP I ligands of CCR4 and  **CCR8** .  Ref: 11560999 J Exp Med, 2001
- T regulatory cells, which inhibit dendritic cell function and are probably involved in the termination of ACD, are sensitive to MCP 1, MIPs and TARC, but express high levels of  **CCR8**  and are more specifically attracted by I 309  [ **CCL1** ] .  Ref: 11876523 Arch Dermatol Res, 2002
- The chemokine receptor  **CCR8**  mediates human endothelial cell chemotaxis induced by I 309  [ **CCL1** ]  and Kaposi sarcoma herpesvirus encoded vMIP I and by lipoprotein a stimulated endothelial cell conditioned medium.  Ref: 11133740 Blood, 2001
- Increased responsiveness of murine eosinophils to MIP 1beta CCL4 and TCA 3  [ **CCL1** ]   **CCL1**  is mediated by their specific receptors, CCR5 and  **CCR8** .  Ref: 12050188 J Leukoc Biol, 2002

**Non-interactive relationship** (e.g. studied together, co-existance, homology, etc.)

- **CCR8** , the  **CCL1**  receptor, was identified on endothelial cells, and  **CCL1**  was found to be a chemoattractant for these cells.  Ref: 12489497 Isr Med Assoc J, 2002
- The aim of our study was to characterize the role of the CC chemokine receptor 8  [ **CCR8** ]   **CCR8** , the only described receptor for  **CCL1** , in the rescue of murine thymic lymphoma cells and murine thymocytes from dexamethasone dex induced apoptosis.  Ref: 12525579 J Leukoc Biol, 2003
- The most active chemokines for resting Th IL 10, in terms of calcium mobilization and in vitro migration, were in order of potency CCL2 monocyte chemoattractant protein 1, CCR2 ligand, CCL4 macrophage inflammatory protein 1beta, CCR5 ligand, CCL3 macrophage inflammatory protein 1alpha, CCR1 5 ligand, CCL17 thymus and activation regulated chemokine, CCR4 ligand,  **CCL1**  I 309  [ **CCL1** ] ,  **CCR8**  ligand, CXCL12 stromal derived factor 1, CXCR4, and CCL11 eotaxin, CCR3 ligand.  Ref: 11145678 J Immunol, 2001
- Th2 lymphocytes, which are critical for allergy, employ the CC chemokine receptors CCR4 and  **CCR8**  with the ligands thymus and activation regulated chemokine TARC, macrophage derived chemokine MDC and I 309  [ **CCL1** ] , respectively.  Ref: 11340325 Int Arch Allergy Immunol, 2001
- I 309  [ **CCL1** ]  and  **CCR8**  mRNAs were not detected in unaffected skin and were up regulated at the skin site of nickel allergic reaction, with an earlier expression kinetics compared with IL 10 and IL 4.  Ref: 11145678 J Immunol, 2001
- Inhibition of I 309  [ **CCL1** ]  receptor  **CCR8**  signaling by pertussis toxin increased the apoptosis rate of ATL cell cultures in the presence and absence of external apoptotic stimuli.  Ref: 11493464 Blood, 2001
- Such pattern of  **CCL1**  responsiveness suggests that the  **CCR8**   **CCL1**  axis may regulate trafficking of cutaneous Treg and memory T cells into the skin.  Ref: 12442333 Eur J Immunol, 2002
- We studied the role of the  **CCR8**  ligand,  **CCL1** , in the specific recruitment of Th2 cells and eosinophils to the lung in a murine model of allergic airway disease.  Ref: 12707363 J Immunol, 2003
- **CCR8**  I 309  [ **CCL1** ]  driven recruitment of both resting and activated Th IL 10 cells may be critically involved in the regulation of Th1 mediated skin allergic disorders.  Ref: 11145678 J Immunol, 2001
- Although it has been hypothesized that the rescuing effect of  **CCL1**  from apoptosis could be  **CCR8**  mediated, here, we formally demonstrate the role of such receptor as its selective antagonist encoded by the MC148 gene of molluscum contagiosum virus MC148 vMCC I inhibits v MIP 1 and  **CCL1**  induced rescue activity.  Ref: 12525579 J Leukoc Biol, 2003
- The  **CCR8**  ligand, TCA3  [ **CCL1** ] , is shown to be produced predominantly by resident microglia of the CNS in response to TNF.  Ref: 12471141 J Immunol, 2002
- Using antibodies specific for CCR5 and  **CCR8** , the chemotactic response to MIP 1beta and TCA 3  [ **CCL1** ] , respectively, was reduced significantly.  Ref: 12050188 J Leukoc Biol, 2002
- Here, we show that human peripheral blood CD4 and CD8 cutaneous CLA, but not intestinal memory integrin beta 7 nor IL 4 producing T cells, represent major subpopulations of circulating T cells that specifically migrate in response to the chemokine I 309  [ **CCL1** ]   **CCL1**  by virtue of  **CCR8**  expression.  Ref: 12442333 Eur J Immunol, 2002
- We show here that the  **CCR8**  restricted agonist Kaposi sarcoma associated herpesvirus encoded chemokine viral macrophage inflammatory protein 1 vMIP 1 rescues thymic lymphoma cells from dex induced apoptosis, similar to  **CCL1** ,.  Ref: 12525579 J Leukoc Biol, 2003
- Additionally, increased expression of the  **CCR8**  ligand, TCA 3  [ **CCL1** ] , has been detected in affected lungs in a mouse model of asthma.  Ref: 12496446 J Immunol, 2003
- Using  **CCR8**  mice, evidence is provided that TCA3  [ **CCL1** ]   **CCR8**  interactions contribute to rapid onset CNS inflammation.  Ref: 12471141 J Immunol, 2002
- Immunohistochemical studies identified  **CCR8**  and I 309  [ **CCL1** ]  on the endothelium of human atherosclerotic plaques and in endothelial derived spindle cells of Kaposi sarcoma.  Ref: 11133740 Blood, 2001
